# 3 directional Inception-ResUNet: Deep spatial feature learning for multichannel singing voice separation with distortion

**DOI:** 10.1371/journal.pone.0289453

**Published:** 2024-01-29

**Authors:** DaDong Wang, Jie Wang, MingChen Sun

**Affiliations:** 1 School of Mathematics and Computer Science, Jilin Normal University, Siping, Jilin, China; 2 School of Computer Science and Technology, Jilin University, Changchun, Jilin, China; Anhui University, CANADA

## Abstract

Singing voice separation on robots faces the problem of interpreting ambiguous auditory signals. The acoustic signal, which the humanoid robot perceives through its onboard microphones, is a mixture of singing voice, music, and noise, with distortion, attenuation, and reverberation. In this paper, we used the 3D Inception-ResUNet structure in the U-shaped encoding and decoding network to improve the utilization of the spatial and spectral information of the spectrogram. Multiobjectives were used to train the model: magnitude consistency loss, phase consistency loss, and magnitude correlation consistency loss. We recorded the singing voice and accompaniment derived from the MIR-1K dataset with NAO robots and synthesized the 10-channel dataset for training the model. The experimental results show that the proposed model trained by multiple objectives reaches an average NSDR of 11.55 dB on the test dataset, which outperforms the comparison model.

## 1 Introduction

Music training implemented by robots is more interesting than other devices. The robot must complete accompaniment, intelligent music synthesis, interactive scoring, singing voice separation,and lyric synchronization. Singing voice separation is the basis of other functions and is also important for improving robot speech recognition accuracy. Robots usually have 2–4 microphones. Recently, an increasing number of studies have focused on exploring multimicrophone source separation in real-world applications [1]. Due to their physiological structure, humans can easily distinguish between singing voice and instrumental accompaniment when listening to a song. However, this is challenging for machine or deep learning models since singing voice and accompaniment are strongly correlated in time and frequency. Moreover, multichannel singing voice separation is more challenging due to model complexity, background noise, microphone distortion, and other factors.

There are two major approaches for multichannel source separation in the early stages: microphone array processing and blind source separation (BSS) [2]. The BSS approaches usually exploit the statistical characteristics of the mixture of the singing voice, accompaniment, and noise, while the microphone array processing approaches usually concern the signal models. The ideas between the two major approaches often borrow from each other. Recently, supervised singing voice separation using deep neural networks (DNNs) has received widespread attention from researchers with great success [3]. Typically, these methods [4, 5] learn a mapping function from singing voice features to separation targets through supervised learning algorithms. Compared to signal models, deep learning can automatically extract the most powerful singing voice features in the mixture. The deep learning models can process the original high-dimensional data without knowledge requirements for feature design, mine the structured features in the singing voice, and output the structured prediction. Using DNNs in training has emerged as a promising trend in microphone array processing and BSS [6, 7].

Researchers have proposed many deep learning models for BSS, including recurrent neural networks (RNNs) [8], convolutional neural networks (CNNs) [9], U-Nets [4, 10, 11], long short-term memory (LSTM) [12], generative adversarial networks (GANs) [13, 14], etc. The results show that a DNN model trained with the singing voices of dozens of singers can separate the singing voices of others. Most DNN models [12, 14] deal with the time-frequency (T-F) domain spectrogram generated from the short-term Fourier transform (STFT). DNN models extract the spectral characteristics of the singing voice and accompaniment.

Microphone array processing approaches traditionally, utilize spatial cues such as geometry-based information to construct signal models [15]. In recent years, DNNs have emerged in multimicrophone array processing approaches for parameter estimation [16] and spatial and spectral feature extraction [7, 17]. Joint modeling of spatial and spectral information potentially improves the separation performance [2]. However, most previous DNN-based approaches do not fully utilize the spatial and spectral information of the spectrogram or lose part of the information in training, which leads to a certain residual noise in the separation results. Additionally, selecting a proper training target for singing voice separation is difficult. Single-objective loss, such as MSE or L1 loss, converges faster; however, the results are not necessarily the best, and the problem with multiobjective training is balancing multiple objectives. The code and data are available on GitHub (https://github.com/sheiaaaa/geshengfenli).

Our contributions include three aspects:

- We propose a 3-directional Inception-ResUNet framework for improving the utilization of spatial and spectral information of the spectrogram.
- We design a joint training objective strategy for obtaining better separation performance, which includes magnitude consistency loss, phase consistency loss and magnitude correlation consistency loss.
- We construct a 10-channel dataset that can be used to test multichannel singing voice separation algorithms.

## 2 Related work

### 2.1 General training target

In the context of sound source separation and localization, the combined information from interchannel level differences (ILDs), interchannel phase differences (IPDs) [18], and spectrograms can be effectively leveraged using spectral magnitude masking (SMM) [19], Phase-sensitive masking (PSM) [20], or complex ideal ratio masking (cIRM) [21] as the training objectives. By employing these techniques, it is possible to improve the performance of sound source separation algorithms and achieve more accurate localization results. In general, the STFT spectrogram expressed in complex numbers consists of two kinds of information: magnitude and phase. As the training data must be scalar, some studies use magnitudes, namely, the modulus of complex numbers. Jansson et al. [4] components an audio signal by converting it to an image, processing it with a U-Net neural network, and storing the resulting spectral mask. GNU-Net [10] leverages a supervised symmetric encoder-decoder architecture for generating full-resolution feature maps. SVSGAN [14] leverages the generative adversarial network with a time-frequency masking function for singing voice separation. SMM [19] involves masking the spectrogram based on the energy distribution across frequency bins, allowing for better separation of the target sources. The spectral magnitude mask training target can be defined as the magnitude of the clean singing voice divided by that of the mixture. PSM [20] extends the SMM by multiplying *cosθ*, where *θ* denotes the difference between the clean singing voice and the mixture phase. It focuses on preserving the phase information in the separated signals, ensuring that the localization accuracy is maintained. Complex ideal ratio masking (cIRM) [21] is a more advanced technique that combines both spectral and phase information to generate masks that improve the quality of the separated signals. In addition, compared to monaural singing voice separation, multichannel singing voice separation can use spatial in addition to spectral information. The ILD and IPD can be used in training. Yilmaz et al. [18] proposed W-disjoint orthogonality for effectively separating mixture signals. Chen et al. [7] proposed a multichannel learning-based method for sound source separation in a reverberant field. Leglaive et al. [22] designed a probabilistic reverberation method for separating multichannel audio sources.

In summary, by incorporating the ILD, IPD, and spectrogram information and utilizing SMM, PSM, or cIRM as the training targets, it is possible to develop more robust and accurate sound source separation and localization algorithms. This can be particularly useful in applications such as audio postprocessing, music production, and audio enhancement, where the ability to separate and localize sound sources accurately is crucial for achieving optimal results.

### 2.2 Deep learning singing voice separation

Multichannel singing voice separation is a regression problem. Recently, most methods have adopted an encoder-decoder structure to solve this problem. The encoder structure typically uses convolution and pooling to extract spectral features of the clean singing voice from the mixture of the ILD, IPD, and spectrogram, while the decoder structure uses deconvolution to recover the spectrogram of the clean singing voice. As downsampling yields detail loss, upsampling is usually compensated with skip connections that connect the spectrogram with the result of upsampling in the same layer. Since Wang et al. used a 4-layer DNN to separate sources [23], dozens of methods for singing voice separation using DNNs, such as CNNs, RNNs, and various variants, have been proposed [3]. Stoter et al. used three bidirectional LSTMs to compose a benchmark system on the MUSDB18 dataset [24]. After Jansson et al. used U-Net for singing voice separation that surpassed the previous methods [4, 11], a few improved versions based on U-Net architecture achieved better performance. Qian et al. used stripe-transformer blocks to learn the deep stripe feature in encoder and decoder blocks, which are composed of residual CNN blocks [5]. Geng et al. developed a gated nested U-Net(GNU-Net) architecture to generate full-resolution feature maps [10]. Yuan et al. used genetic algorithms to search the effective MRP-CNN structures, which are composed of various-sized pooling operators, to extract multiresolution features. [25]. The above methods are spectrogram-based methods with better performance than U-Net. Simon et al. use a hybrid model in the newest Demucs system. The hybrid model has a parallel time branch in addition to the spectrogram branch [26]. Kong et al. constructed a residual U-Net architecture with a time branch and a spectrogram branch and estimated the phase by cIRMs [27]. The above two methods combine spectrogram and time encoding and decoding structures, which significantly improve the separation performance on the MUSDB18 dataset. However, when separating singing voices accompanied by noise and distortion, the separation performance of all the above methods is significantly degraded.

In summary, the U-shaped encoding and decoding network was adopted for singing voice separation. Adding components that improve network performance to downsampling and upsampling can improve the separation performance. The combination of spectrogram and time can achieve better results. All of the models mentioned above were trained on datasets without distortion.

### 2.3 Robot music accompaniment studies

With the rapid development of robot technology, increasing attention has been given to the combination of robots and music composition. As an interdisciplinary field, robot music accompaniment studies have attracted the attention of computer scientists, musicians, and artists, as well as bringing new possibilities for robot applications and music education. In this field, many researchers have achieved significant results, including the development of algorithms that can automatically create music [28], the combination of robots and musical instruments to achieve human‒machine collaboration [29], and the design of intelligent systems that can understand music and dance [30]. PepperOSC [31] connects the Pepper and NAO robots by leveraging sound production tools, which improves the effectiveness and attractiveness of human-robot interaction. Pluta et al. [32] leveraged a robot to explore the re-excitation of an acoustic guitar string and improved a simple synthesis model of a vibrating string based on the finite difference method. Wang et al. [33] effectively combined music and robots to make the robot accurately express music in real-time. Engstrom et al. [34] designed a robot application to play drums in rhythm to an external audio source. Qin et al. [35] developed a humanoid robot dance system driven by musical structures and emotions. Okamoto et al. [36] proposed a dancing robot system, that can make the robot listen to and dance along with musical performances. Bando et al. [37] explored sound source localization and separation in robots. Chu et al. [38] proposed a deep learning-based method to identify musical beats and styles to construct a human dancing robot. Byambatsogt et al. [39] presented a multitask learning-based model for a guitar chord recognition. Jung et al. [40] proposed a music therapy robot to alleviate depressive emotions.

In summary, robot music accompaniment studies have achieved remarkable results for algorithm development, and human-machine collaborative performance. Robot music accompaniment studies not only enrich the possibilities of robot applications and music education but also provide us with new perspectives to understand and explore music composition and performance.

## 3 Problem statement and formulation

Most DNN-based singing voice separation often consists of three stages [10, 12, 14]

- **Time-frequency transformation**. The time domain signals of the singing voice and mixture are decomposed into two-dimensional time-frequency-domain spectrograms by STFT.
- **Construct the separation model**. The model output is a soft mask that separates the mixture spectrogram into a singing voice and a nonvoice spectrogram.
- **Frequency-time transformation**. The target singing voices in the time domain are reconstructed from the mixture spectrogram multiplied elementwise with the mask by inverse short-time Fourier transform (ISTFT).

The time domain mixture signal gathered by the *i*th microphone can be defined as follows:

$$\begin{array}{c} x_{i}\left(t\right)=\sum_{k=1}^{N}\sum_{l}h_{ik}\left(l\right)s_{k}\left(t-l\right) \end{array}$$ (1)

where *N* denotes the number of sources, *M* denotes the number of microphones, *k* ∈ {1, 2, …, *M*;*k* ≠ *i*}, *s*_*k*_(*t*) denotes the signals recorded by the *i*th microphone from the *k*th source, *h*_*ik*_(*l*) denotes the impulse response from the *k*th source to the *i*th microphone, and *l* denotes the impulse index. The spectrogram at time-frequency point (*t*, *f*) of *x*_*i*_(*t*) can be approximated as [41]

$$\begin{array}{c} x_{i}\left(t,f\right)\approx \sum_{k=1}^{N}h_{ik}\left(f\right)s_{k}\left(t,f\right) \end{array}$$ (2)

where *h*_*ik*_(*f*) denotes the frequency response from the *k*th source and *s*_*k*_(*t*, *f*) is the STFT of *s*_*k*_(*t*). Since multiple noise sounds can be modeled as a single source [2], we denote with ${\bar{S}}_{M}\left(t,f\right)$, ${\bar{S}}_{V}\left(t,f\right)$ and ${\bar{S}}_{N}\left(t,f\right)$ the spectrogram of music, singing voice and noise recorded by the *i*th microphone, respectively. *x*_*i*_(*t*, *f*) can be described by,

$$\begin{array}{c} x_{i}\left(t,f\right)\approx {\bar{S}}_{iM}\left(t,f\right)+{\bar{S}}_{iV}\left(t,f\right)+{\bar{S}}_{iN}\left(t,f\right) \end{array}$$ (3)

where *x*_*i*_(*t*, *f*) denotes the spectrogram of *x*_*i*_(*t*) and ${\bar{S}}_{ik}\left(t,f\right)$ represents *h*_*ik*_(*f*)*s*_*k*_(*t*, *f*).

Taking the *i*th microphone as a reference, we used two relative transfer functions between the *i*th microphone and the *k*th microphone [15]. The *i*th microphone and the *k*th microphone ILDs are defined as

$$\begin{array}{c} ILD_{ik}\left(t,f\right)=20log\frac{|x_{k}\left(t,f\right)|}{|x_{i}\left(t,f\right)|} \end{array}$$ (4)

The *i*th microphone and the *k*th microphone phase differences (IPDs) are calculated as

$$\begin{array}{c} IPD_{ik}\left(t,f\right)=e^{j(∠x_{k}\left(t,f\right)-∠x_{i}\left(t,f\right)} \end{array}$$ (5)

where ∠ denotes the phase in radians of a complex number.

We concatenate the spatial cues *ILD* within the real component of *IPD* and the imaginary component of *IPD*. Subsequently, we leverage the spectral features of each microphone to form the input features, which can be defined as follows:

$$\begin{array}{c} {\left\{ILD_{ik}\left(t,f\right),IPD_{ik}\left(t,f\right).real,IPD_{ik}\left(t,f\right).imag,|x_{k}\left(t,f\right)|\right\}}_{k=1}^{M} \end{array}$$ (6)

where *IPD*(*t*, *f*).*real* denotes the real component of *IPD* and *IPD*(*t*, *f*).*imag* denotes the imaginary component of *IPD*.

The prediction targets ${\hat{Y}}_{iV}\left(t,f\right)$ are the magnitude spectrogram of the singing voice. After training, the DNN model’s output predictions, which are a time-frequency mask, can predict the magnitude spectrogram of the target singing voice from the multichannel spectrogram. The mask [20] can be defined as

$$\begin{array}{c} m1\left(t,f\right)=\frac{|{\hat{Y}}_{iV}\left(t,f\right)|}{|X_{i}\left(t,f\right)|}cos\left(\theta \right) \end{array}$$ (7)

$$\begin{array}{c} m2\left(t,f\right)=\frac{|{\hat{Y}}_{iV}\left(t,f\right)|}{|X_{i}\left(t,f\right)|}sin\left(\theta \right) \end{array}$$ (8)

where *X*_*i*_(*t*, *f*) denotes the spectrogram of the reference microphone, *f* = 1, 2, 3, …, *F* denotes different frequencies, and *θ* denotes the difference between the predicted singing voice phase and mixture phase of the reference microphone. We apply the soft mask to *X*_*i*_ to estimate the predicted separation spectrogram ${\hat{Y}}_{iV}$, which can be defined as follows:

$$\begin{array}{c} {\hat{Y}}_{iV}\left(t,f\right).real=|X_{i}\left(t,f\right)|⊗\left(m1\left(t,f\right)⊗cos\left(∠x_{i}\left(t,f\right)\right)-m2\left(t,f\right)⊗sin\left(∠x_{i}\left(t,f\right)\right)\right) \end{array}$$ (9)

$$\begin{array}{c} {\hat{Y}}_{iV}\left(t,f\right).imag=|X_{i}\left(t,f\right)|⊗\left(m1\left(t,f\right)⊗sin\left(∠x_{i}\left(t,f\right)\right)+m2\left(t,f\right)⊗cos\left(∠x_{i}\left(t,f\right)\right)\right) \end{array},$$ (10)

where ∠ denotes the phase in radians of a complex number, *x*_*i*_(*t*, *f*) denotes the spectrogram of *x*_*i*_(*t*), ⊗ stands for elementwise operation, ${\hat{Y}}_{iV}\left(t,f\right).real$ denotes the real component of *Y*_*iV*_(*t*, *f*) and *Y*_*iV*_(*t*, *f*).*imag* denotes the imaginary component of *Y*_*iV*_(*t*, *f*).

### 3.1 Overall architecture

**Fig 1:**
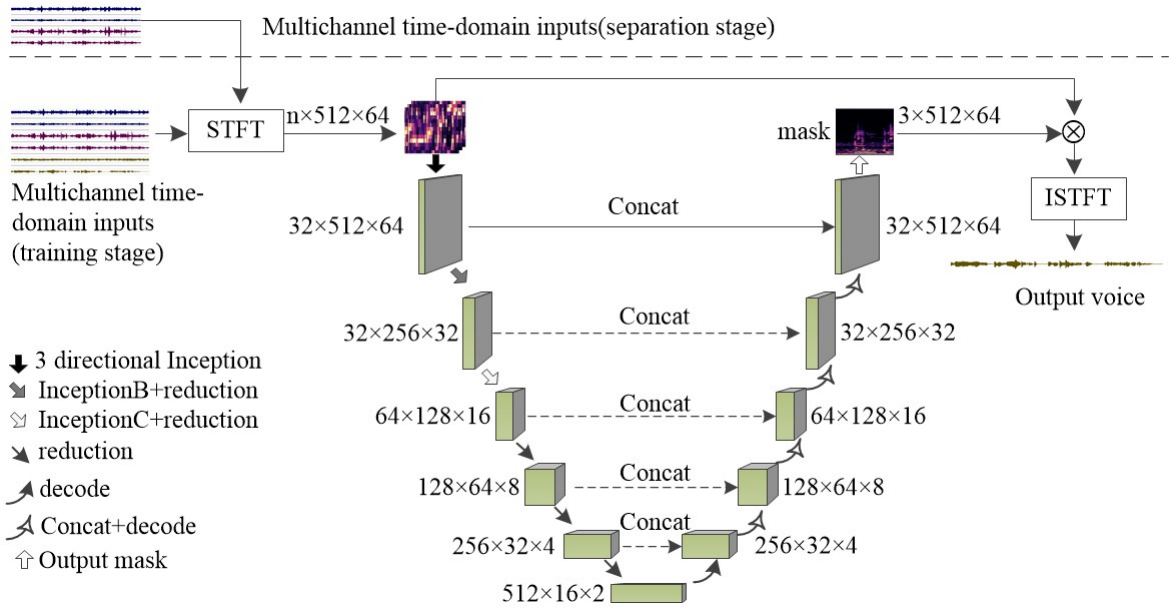
Proposed model.

The proposed model is shown in Fig 1. It consists of 6 encoder/decoder layers. The first encoder layer consists of 3 directional Inception- ResNet blocks. Both the second and third encoder layers consist of an Inception-ResNet block and a reduction block. The fourth and fifth encoder layers consist of a reduction block. In each decoder layer, we first used a fractionally strided convolution with stride 2 and kernel size 2×2, batch normalization, and LeakyReLU, then used two convolutions with stride 1 and kernel size 3×3, batch normalization, and LeakyReLU, which was followed by 4 Inception-ResNet blocks. In the final layer, we used 1 × 1 convolutions and a sigmoid activation function to output a 1-channel mask. The mask consists of three submasks: $|{\hat{Y}}_{iV}\left(t,f\right)|/|X_{i}\left(t,f\right)|$, *cos*(*θ*), and *sin*(*θ*).

## 4 Inception-ResUNet framework

In our approach, the songs are recorded at a sample rate of 16,000 Hz. We leverage a 1024-point window size and a 512-point hop size in STFT. Namely, the time window is 64 ms, and the overlap between two consecutive windows is 32 ms. Thus there are 32 time windows in 1 s. Each window is transformed by STFT, generating complex coefficients of 512 valid positive frequency channels between 0 and 8,000 Hz. Therefore, the signal lasting 2 s will be transformed to a 512 × 64 spectrogram.

### 4.1 Multichannel singing voice alignment

**Fig 2:**
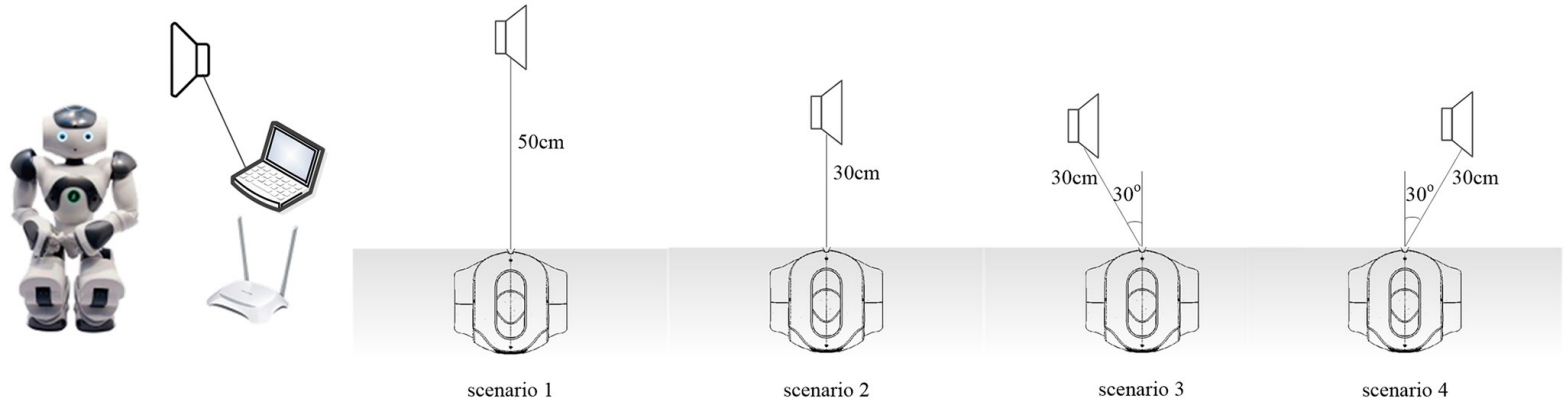
Recording scenarios.

The recording scenarios are shown in Fig 2. The recording equipment includes a computer, a robot, and an external speaker. The computer connects the NAO robot via a wireless network. The external speaker that plays the singing voice is placed in front of the robot’s head. The computer plays the singing voice. The robot plays the accompaniment and records the mixture.

There are three types of delay during recording: network transmission delay, sound propagation delay, and processing delay. As the singing voice and the accompaniment are played on the computer and the robot, respectively, the sound propagation delay of the two sources is different. When recordings, singing voices, and accompaniments are combined into a training dataset, they must be aligned.

Let *CR*_*i*_ denote the *ith* channel recordings, and *V* denote the singing voice, which can be defined as follows:

$$\begin{array}{c} \begin{array}{cc} CR_{i} & =\{cr_{i}^{0},cr_{i}^{1},cr_{i}^{2},\cdots ,cr_{i}^{\left(N-1\right)}\},\\ V & =\{v_{0},v_{1},v_{2},\cdots ,v_{\left(M-1\right)}\} \end{array} \end{array}$$ (11)

where |*CR*_*i*_| = *N* − 1, |*V*| = *M* − 1, $cr_{i}^{j}$ denotes the *j*th data sample in the *i*th channel recording and *v*_*k*_ denotes the *k*th original value in the singing voice. Subsequently, we slice *CR*_*i*_ and *V* for singing voice alignment, which is defined as follows:

$$\begin{array}{c} \begin{array}{cc} F_{CR_{i}}^{p} & =\{cr_{i}^{p},cr_{i}^{p+1},cr_{i}^{p+2},\cdots ,cr_{i}^{p+L}\},\\ F_{V}^{q} & =\alpha \{v_{q},v_{q+1},v_{q+2},\cdots ,v_{q+L}\} \end{array} \end{array}$$ (12)

where $F_{CR_{i}}^{p}$ denotes the recording fragment starting with *p*, $F_{V}^{q}$ denotes the singing voice fragment starting with *q*, *L* is the sliding window size and *α* ∈ [0, 1] denotes the adjustable coefficient. When $F_{CR_{i}}^{p}$ and $F_{V}^{q}$ are aligned, *p* and *q* are calculated as follows:

$$\begin{array}{c} \text{arg}\underset{p=L/8\;0\leq q\leq L/4}{max}\frac{E(\left(F_{CR_{i}}^{p}-E\left(F_{CR_{i}}^{p}\right)\right)\left(F_{V}^{q}-E\left(F_{V}^{q}\right)\right))}{\sqrt{D(F_{CR_{i}}^{p})}\sqrt{D(F_{V}^{p})}} \end{array}$$ (13)

where *E* is the mathematical mean, and $D(F_{CR_{i}}^{p})$ and $D(F_{V}^{q})$ are the standard deviation of $F_{CR_{i}}^{p}$ and the standard deviation of $F_{V}^{q}$,respectively.

### 4.2 3 directional Inception-ResNet blocks

**Fig 3:**
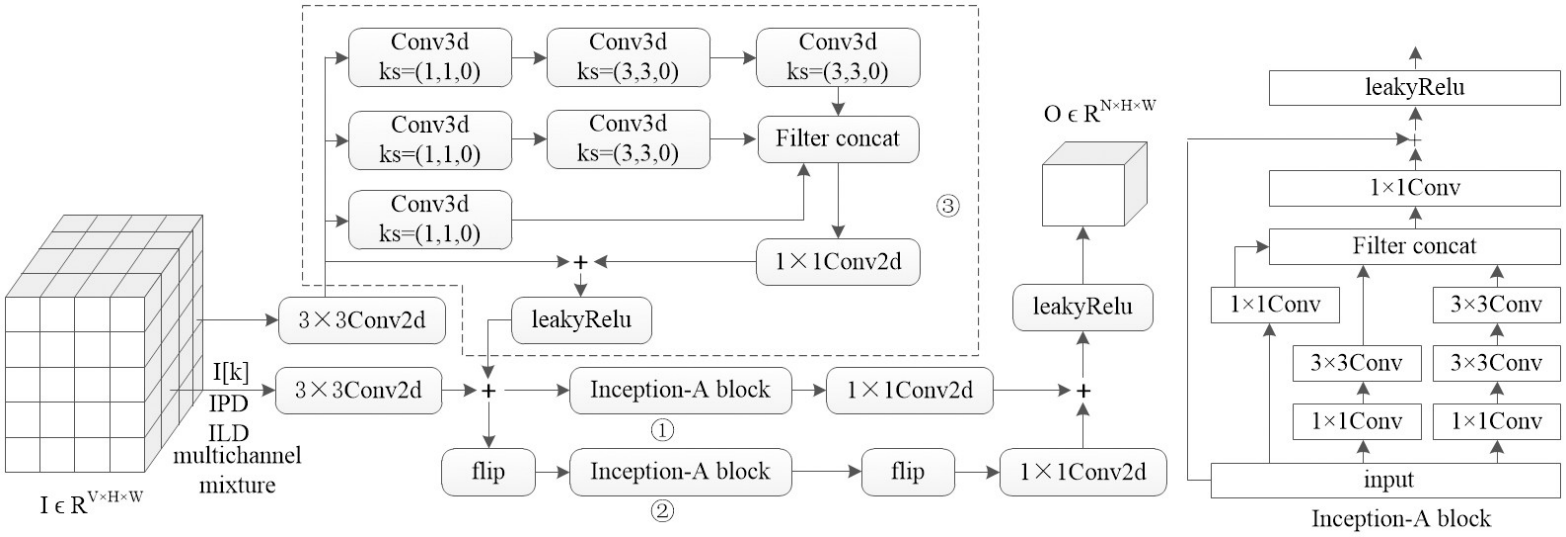
3-directional Inception-ResNet block.

The detailed structure of the 3 directional Inception-ResNet blocks is shown in Fig 3. The implementations of the two horizontally oriented Inception-ResNet blocks are similar to that of [42, 43], and each 5 × 5 2D convolution is replaced by two 3 × 3 convolutions The flip block in Fig 3 represents the flipping operation of the spectrogram in the horizontal direction. We used a scaling factor of 0.17 to scale the residuals. Ten iterations of the InceptionA block were used in the horizontal direction to cover the spectrogram. The vertically oriented Inception-ResNet block, which is implemented by 3D convolution to extract the spatial features of the multichannel spectrogram, also includes 3 branches: 1×1 convolution, 3×3 convolution, and 5×5 convolution.

### 4.3 Inception and reduction layers

**Fig 4:**
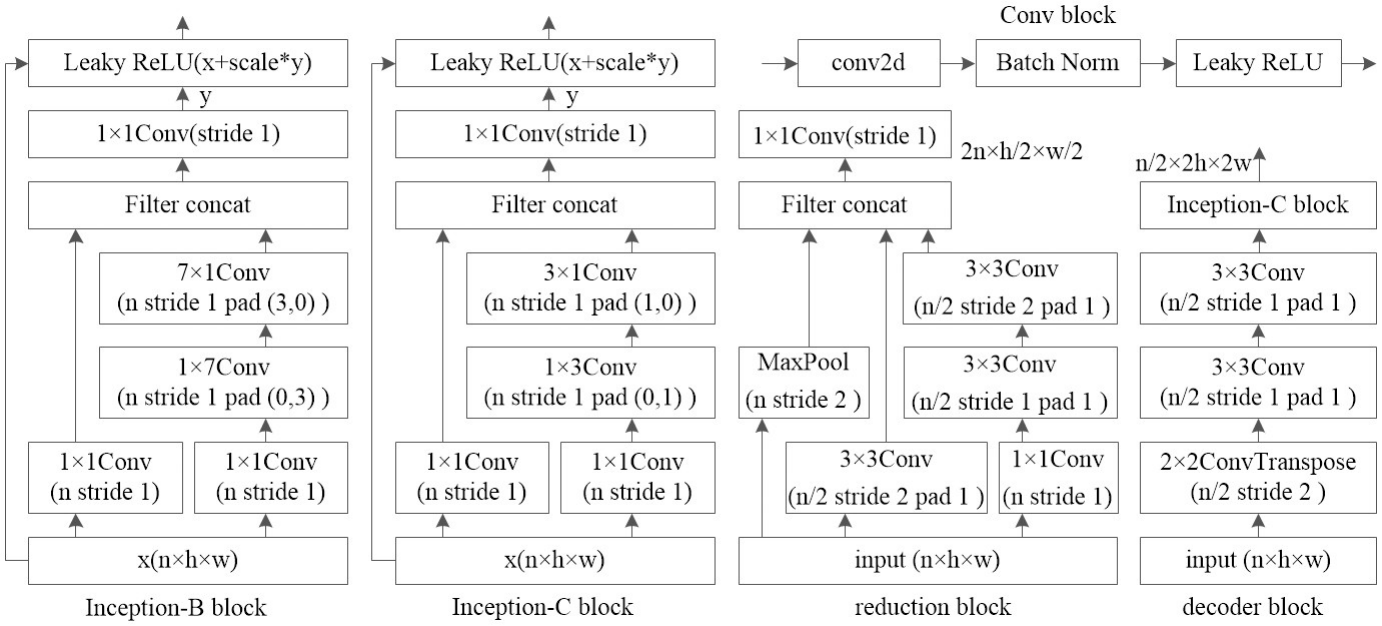
The structure of Inception-B, Inception-C, reduction, and decoder blocks.

The Inception-ResNet blocks used in the second and third encoder layers are shown in Fig 4. Each block is followed by a reduction filter. To reduce the computational cost, we use a 1 × n convolution and an n × 1 convolution to replace a n×n convolution in the Inception-B and Inception-C blocks. In the Inception-B and Inception-C blocks, a 1 × 7 convolution followed by a 7 × 1 convolution and a 1 × 3 convolution followed by a 3 × 1 convolution are used to replace 7 × 7 convolution and 3 × 3 convolution, respectively. Each block is iterated 5 times to cover the entire spectrogram. The scaling factor is set to 0.2 in the Inception-B and Inception-C blocks.

Convolutional networks usually use maximum or average pooling operations to reduce the size of the activation map. Maximum and average pooling are fast and memory-efficient but lose some information in the activation map. To avoid a representational bottleneck, a series of pooling methods such as power average pooling, stochastic pooling, local importance Pooling, and soft pooling, have been proposed [44]. Our implementation of the reduction block in each encoder layer is similar to that of [42]. Two parallel 3 × 3 convolutions with stride 2 are concatenated, as shown in Fig 4. One of the reduction blocks expands the filter banks to avoid the representational bottleneck [43].

### 4.4 Overall optimizing objective

To avoid the phase independence of the predicted spectrogram, we use a hybrid phase-dependent loss function to train *m*1(*t*, *f*) and *m*2(*t*, *f*).

(1) The magnitude consistency loss *Loss*_*M*_ can be defined as follows:

$$\begin{array}{c} Loss_{M}=E_{T,F}\left(\right\|{\hat{Y}}_{iV}\left(t,f\right)-|R_{i}\left(t,f\right)\left|\|\right) \end{array}$$ (14)

where *R*_*i*_(*t*, *f*) denotes the normalized spectrogram of the target clean singing voice and $E_{T,F}$ is the mathematical expectation in data domains T and F.

(2) The phase consistency loss *Loss*_*P*_ = *Loss*_*P*1_+ *Loss*_*P*2_ can be defined as follows:

$$\begin{array}{c} \begin{array}{cc} Loss_{P1} & =E_{T,F}\left(\|\right|{\hat{Y}}_{iV}\left(t,f\right)|cos\left(\theta \right)-(R_{i}\left(t,f\right).real\cdot cos\left(∠x_{i}\left(t,f\right)\right)\\ & +R_{i}\left(t,f\right).imag\cdot sin\left(∠x_{i}\left(t,f\right)\right))\|) \end{array} \end{array}$$ (15)

$$\begin{array}{c} \begin{array}{cc} Loss_{P2} & =E_{T,F}\left(\|\right|{\hat{Y}}_{iV}\left(t,f\right)|sin\left(\theta \right)-(R_{i}\left(t,f\right).real\cdot sin\left(∠x_{i}\left(t,f\right)\right)\\ & -R_{i}\left(t,f\right).imag\cdot cos\left(∠x_{i}\left(t,f\right)\right))\|) \end{array} \end{array}$$ (16)

where ∠ denotes the phase in radians of a complex number, *θ* denotes the difference between the predicted singing voice phase and mixture phase of the reference microphone, *x*_*i*_(*t*, *f*) denotes the spectrogram of *x*_*i*_(*t*), *R*_*i*_(*t*, *f*).*real* denotes the real component of *R*_*i*_(*t*, *f*) and *R*_*i*_(*t*, *f*).*real* denotes the imaginary component of *R*_*i*_(*t*, *f*).

(3) Magnitude correlation consistency loss *Loss*_*C*_, which can be defined as follows:

$$\begin{array}{c} Loss_{C}=1-\frac{E({\hat{Y}}_{iV}\left(t,f\right)|R_{i}\left(t,f\right)|)-E\left({\hat{Y}}_{iV}\left(t,f\right)\right)E(|R_{i}\left(t,f\right)\left|\right)}{\sqrt{E\left({\hat{Y}}_{iV}{\left(t,f\right)}^{2}\right)-E^{2}\left({\hat{Y}}_{iV}\left(t,f\right)\right)}\sqrt{E(|R_{i}{\left(t,f\right)|}^{2})-E^{2}\left(\right|R_{i}\left(t,f\right)\left|\right)}} \end{array}$$ (17)

The overall training objective can be defined as follows,

$$\begin{array}{c} Loss=Loss_{M}+Loss_{P}+Loss_{C} \end{array}$$ (18)

where *Loss*_*M*_ denotes the magnitude consistency loss, *Loss*_*P*_ denotes the phase consistency loss, and *Loss*_*C*_ denotes the magnitude correlation consistency loss.

## 5 Experiment

### 5.1 Data description and preprocessing

In our experiment, the NAO robot recorded a 4-channel mixture in which the accompaniment and singing voice were derived from the public MIR-1K dataset. The MIR-1K dataset is composed of 1000 clips segmenting from 110 songs. All clips are sampled at 16,000 Hz. The left and right channels of these clips record the accompaniment and singing voice, respectively. Table 1 shows parameters such as the sample rate, resolution, clip duration, number of clips, number of singers, number of channels, and total duration of the MIR-1K dataset.

**Table 1:** Dataset basic information.

Parameter	MIR-1K Dataset [45]	Our Dataset
Sample Rate	16 kHz	16 kHZ
Resolution	16 bit	16 bit
Clip Duration	4 s-13 s	3 s-11 s
Number of Clips	1,000	2211
Number of Singers	8 females and 11 males	8 females and 11 males
Number of Channels	2	10
Total Duration	99 min	105 min

**Fig 5:**
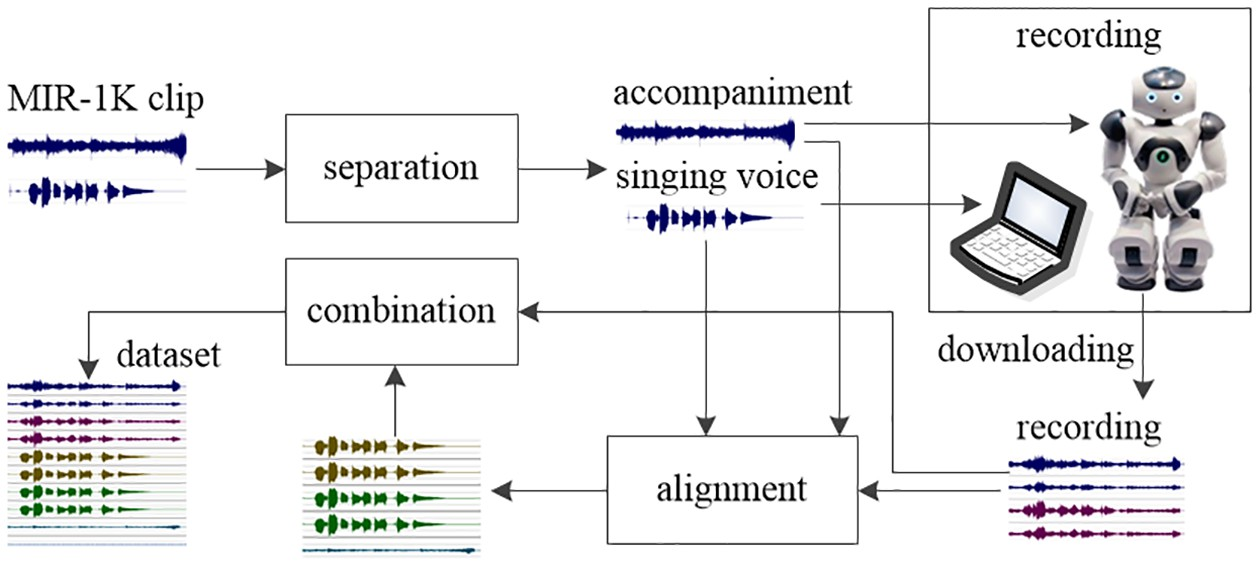
Dataset processing.

The dataset production process consists of five steps: separation, recording, downloading, alignment, and combination, as shown in Fig 5. The stereo clips in the MIR-1K dataset are separated into two monaural clips: the singing voice clip and the accompaniment clip. They are played by the computer and the NAO robot during the recording process. Acoustic signals are gathered by 4 microphones and stored on the NAO robot. The 4-channel recording is aligned with the pure singing voice and accompanied by four aligned monaural singing voice clips and one aligned monaural accompaniment clip. Finally, the 4-channel recording clip, 4 aligned monaural singing voice clips, 1 aligned monaural accompaniment clip, and a monaural noise clip are combined into a 10-channel clip.

In our experiment, the song clips were recorded in an unshielded lab. The background noises also included the noise generated by the fan on the robot’s head. The song clips were sampled at 16000 Hz. *L* was set to 64000. As the distances between the microphones on the robot’s head are less than 12 cm, the delay deviations between the microphones are less than 0.3 ms or 5 sampling times. The experiments showed that in scenario 1, most delay deviations are 3 sampling times. In this paper, we calculated *p* and *q* for each channel. Clips with a *q* deviation between 2 channels exceeding 6 sampling times were discarded. The training dataset included 2,211 aligned 10-channel clips.

**Fig 6:**
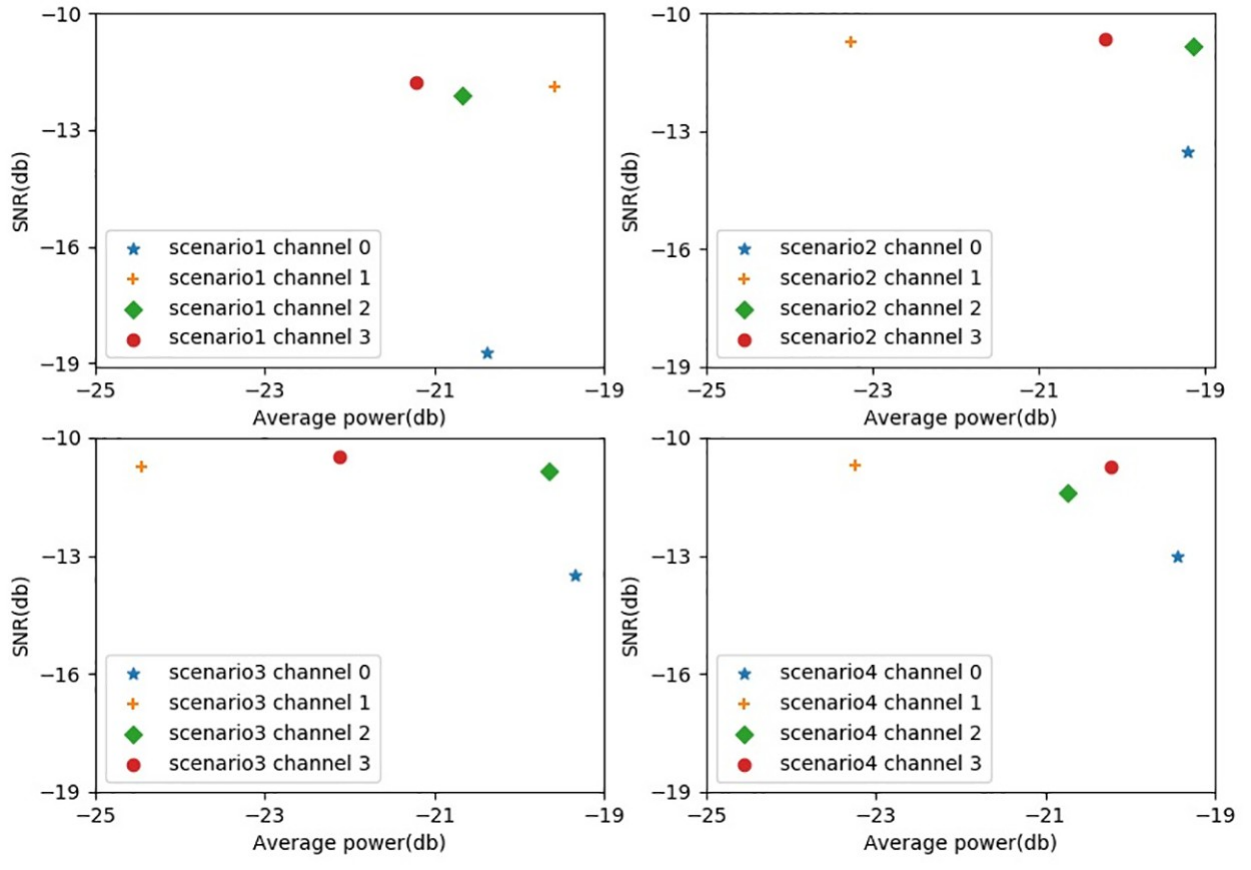
The SNR distribution of the dataset.

The SNR of the singing voice in different scenarios is shown in Fig 6. The mean SNR of the singing voice recorded by the second microphone was the largest. The second microphone was chosen as the reference microphone in our experiment.

### 5.2 Implementations and metrics

In the training stage, the magnitude spectrogram of the mixture was used as the network input, and the magnitude spectrogram of the singing voice was used as the target in the loss function to measure the gap between the predicted result and the singing voice. To evaluate the performance of the proposed model, we trained the proposed model on our dataset. We used 10 Inception-A blocks, 5 Inception-B blocks, and 5 Inception-C blocks in the proposed model. A total of 448 clips were randomly selected in the dataset for training. We randomly selected 448 clips from the 2211 clips for training and 643 clips for testing the performance of the proposed model, and these clips contained each singer’s singing voice in different scenarios. We trained each network for 100 epochs. The optimizer was ADAM. The learning rate was set to 0.00001. The batch size was set to 8. To compare the performance with other models, we also trained the model on the MUSDB18 and MIR-1K datasets for monaural singing voice separation.

To evaluate the quality of separation, the source-to-distortion ratio (SDR), source-to-interferences ratio (SIR), and sources-to-artifacts ratio (SAR) were taken as objective evaluation criteria [46]. A higher value for each ratio indicates better separation. We used the BSS EVAL toolbox to calculate SDR, SIR, and SAR. We also calculated the normalized SDR (NSDR) provided in the BSS EVAL toolbox.

### 5.3 Performance nalysis

The performance of the proposed model was evaluated on our dataset,the MUSDB18 dataset, and the MIR-1K dataset. Table 2 shows the ablation study on the dataset. The model trained with the mixture, IPD, and ILD for the PSM target achieved better performance than the SMM target. The model with the 3DIR blocks has better performance than the others. The performance of the model with 2 directional Inception-ResNet (2DIR) blocks or 1 directional Inception-ResNet (1DIR) block was improved by adding IPD and ILD to the mixture, while the performance improvement was not obvious for the model with 3 directional Inception-ResNet blocks. In other words, the model with 3 directional Inception-ResNet blocks has the ability to extracted the spatial features of the spectrogram.

**Table 2:** Ablation experiment results on the dataset.

Model	target	data	Vocal NSDR	Vocal SIR	Vocal SAR
3DIR blocks	PSM	mixture+IPD+ILD	11.5462	24.2912	10.6632
3DIR blocks	SMM	mixture+IPD+ILD	11.3162	23.4763	10.4743
3DIR blocks	SMM	mixture	11.3171	23.4681	10.4778
2DIR blocks (① + ③)	SMM	mixture+IPD+ILD	11.1824	23.8703	10.3046
2DIR blocks(① + ②)	SMM	mixture	11.0004	22.4257	10.2268
1DIR blocks(①)	SMM	mixture+IPD+ILD	11.13554	22.5646	10.3663

**Fig 7:**
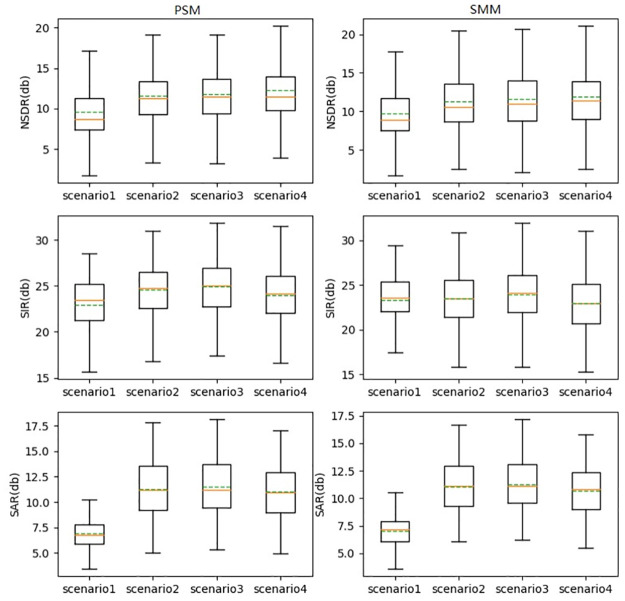
Separation performance of the model.

The separation results for the four scenarios are shown in Fig 7. The means of NSDR and SAR in scenario 1 are the lowest. The main reason is that the SNR in scenario 1 is the lowest. The mean NSDR, SIR, and SAR for the other three scenarios were relatively close. The results show that the model can effectively separate the singing voice in different directions.

Table 3 shows the separation performance on the dataset with different objectives. We can see that the model trained with the *Loss*_*M*_, *Loss*_*P*_and *Loss*_*C*_ objectives achieved higher NSDRs, SIRs, and SARs than the two objectives. The *Loss*_*C*_ objective significantly improved the mean NSDR and SAR on the dataset. Table 4 compares the separation performance of our proposed model with the U-Net and Demucs models. Both models were trained on SMM. The U-Net model was trained with a *Loss*_*M*_ objective like [4]. The results show that the proposed model achieved higher NSDR, SIR, and SAR than U-Net and Demucs. Due to the distortion of the singing voice recorded by a robot, the Demucs model, which was good at separating the undistorted singing voice, did not achieve a higher separation performance.

**Table 3:** Singing voice mean score on the dataset with different objectives.

Objective	Vocal NSDR	Vocal SIR	Vocal SAR
*Loss*_*M*_ + *Loss*_*P*_ + *Loss*_*C*_	11.3171	23.4681	10.4778
*Loss*_*M*_ + *Loss*_*P*_	9.3305	23.2536	8.4747

**Table 4:** Singing voice mean score on the dataset for different models.

Model	Vocal NSDR	Vocal SIR	Vocal SAR
Inception-ResUnet	11.3171	23.4681	10.4778
U-Net [4]	6.5867	17.4178	5.9888
Demucs(V4.0.1) [26]	10.0492	7.2585	7.7515

To compare the performance of Inception-ResUNet with other models, we also trained the Inception-ResUNet model on the MUSDB18 dataset and MIR-1K dataset with 2 directional Inception-ResNet blocks, PSM, and *Loss*_*M*_ + *Loss*_*P*_. Table 5 shows the comparison of Inception-ResUNet with other models for SDR, SIR, and SAR on the MUSDB18 dataset.

As shown in Table 5, the proposed model achieved 7.85 dB on the vocal SDR category and 13.66 dB on the accompaniment SDR category on the MUSDB18 dataset, which outperforms Open Umix, E-MRP-CNN, and D3Net. As shown in Table 6, the proposed model achieves 12.73 dB on the vocal NSDR category and 12.53 dB on the accompaniment NSDR category on the MIR-1K dataset, which outperforms E-MRP-CNN, U-Net, and RPCA-DRNN.

**Table 5:** Comparison of SDR, SIR and SAR of other methods and Inception-ResUNet on the MUSDB18 dataset.

Method	Vocal SDR	Vocal SIR	Vocal SAR	Acc SDR	Acc SIR	Acc SAR
Open Umix [24]	5.57	12.19	5.98	11.66	19.62	12.54
E-MRP-CNN [25]	6.36	13.68	6.60	12.99	16.18	14.41
D3Net [47]	7.24	-	-	13.52	-	-
Inception-ResUNet	7.85	18.75	8.36	13.66	19.74	15.17

**Table 6:** Comparison of NSDR, SIR and SAR of other methods and Inception-ResUNet on the MIR-1K dataset.

Method	Vocal NSDR	Vocal SIR	Vocal SAR	Acc NSDR	Acc SIR	Acc SAR
RPCA-DRNN [48]	8.46	13.75	10.83	8.02	12.32	11.99
U-Net [4]	8.94	14.12	11.40	9.22	14.88	11.19
E-MRP-CNN [25]	10.55	11.16	13.65	11.89	17.80	13.60
Inception-ResUNet	12.73	20.54	14.09	12.53	18.10	14.18

**Fig 8:**
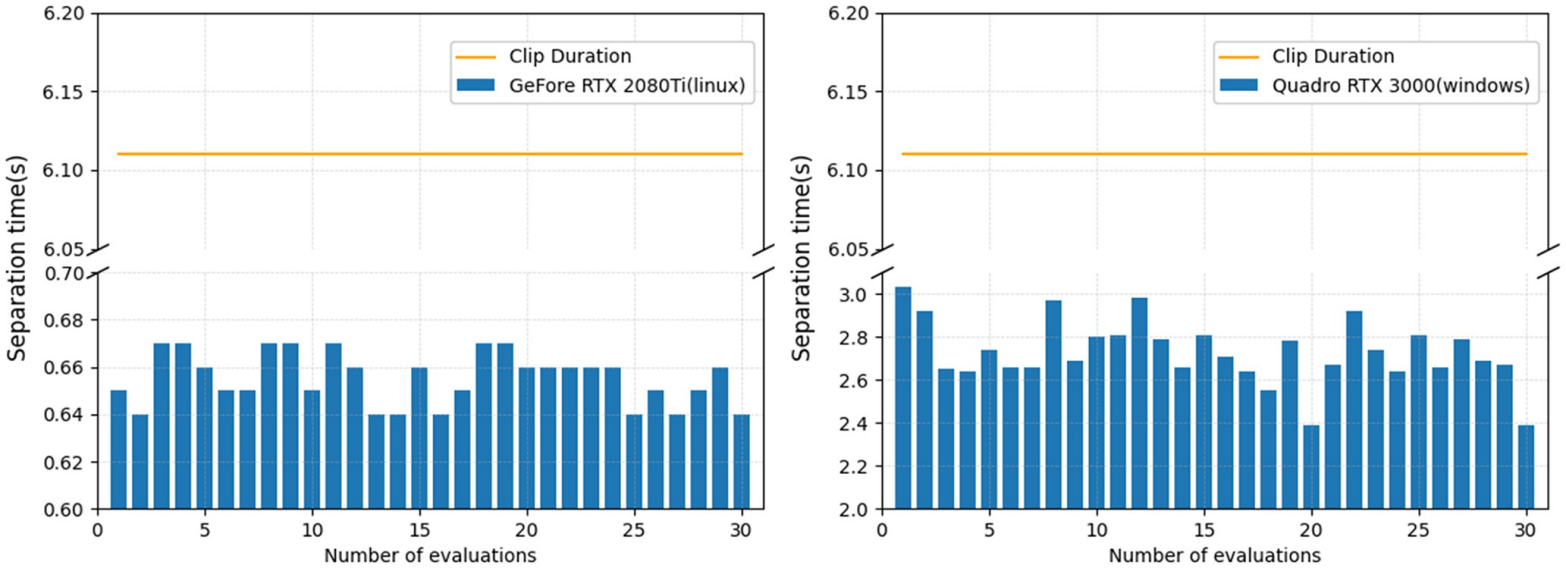
Average processing time (s) on different GPUs of clip “amy_4_02”.

The results of the real-time performance evaluation of the model are shown in Fig 8. A clip with a duration of 6.11 seconds was separated 30 times on two different GUPs. The processing time on GeForce RTX 2080Ti (Linux) was less than 0.68 seconds and less than 3.0 seconds on Quadro RTX 3000 (Windows), both of which were much shorter than the duration of the clip.

## 6 Discussion

**Fig 9:**
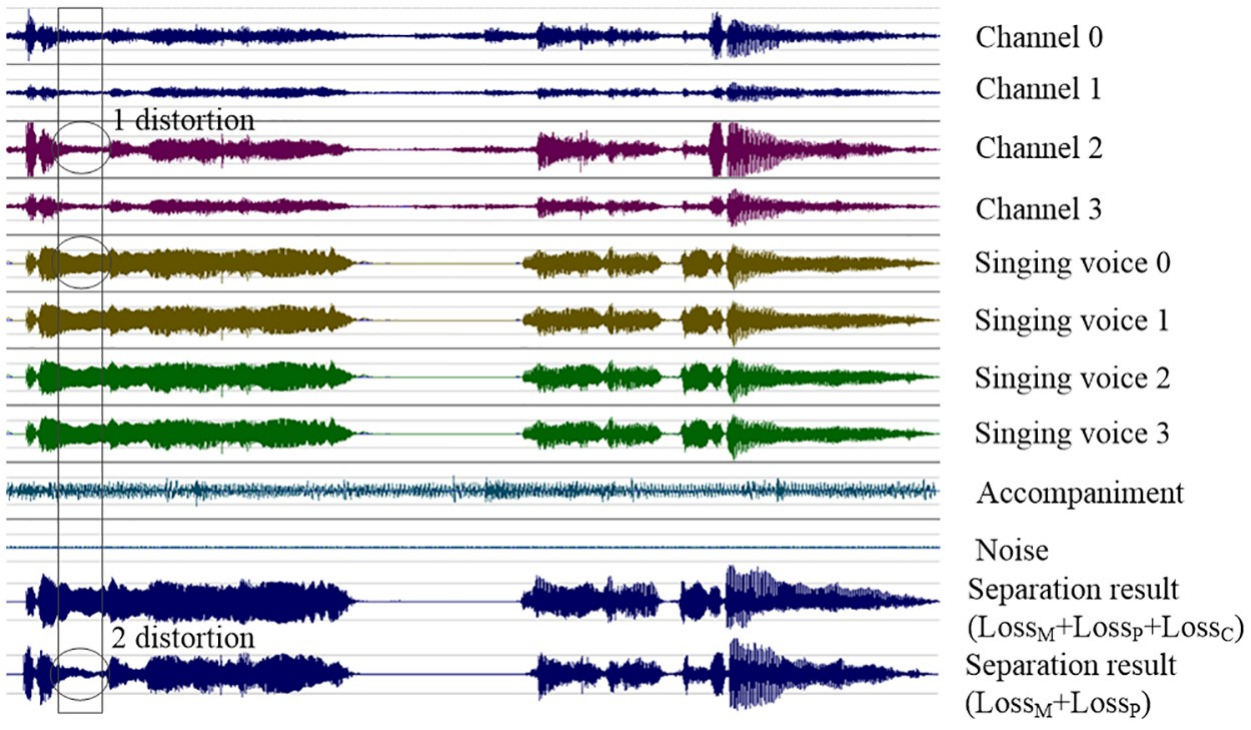
*Loss*_*C*_ corrects the distortion of singing voice separation.

The separation performances of most singing voice separation methods for monaural recordings degrade for separating distorted singing voices. The main reason is that the distortion of the spectrogram is also proportionally reserved. Unfortunately, the multichannel mixture recorded by ordinary robots was distorted, as shown in position 1 in Fig 9. The model trained by *Loss*_*M*_ and *Loss*_*P*_ [4, 25, 49] preserved the distortion, as shown in position 2 in Fig 9. When a model was trained on multiple objectives, improvements in one objective degraded the others. The proposed model trained by *Loss*_*M*_ + *Loss*_*P*_ + *loss*_*C*_ did not achieve the best separation performance on the MUSDB18 dataset. Our experiments showed that a model trained by *Loss*_*M*_ + *Loss*_*P*_ + *loss*_*C*_ had a lower SDR. However, when *Loss*_*C*_ was used to separate distorted singing voices, *Loss*_*C*_ can significantly improved separation performance. *loss*_*C*_ reduced the distortion of the singing voice, and the benefits outweighed the reduction.

## 7 Conclusion

In this paper, we proposed a novel model, 3D Inception-ResUNet, for separating the multichannel singing voice with distortion. We trained the proposed model with multiple objectives: magnitude correlation consistency loss, magnitude consistency loss, and phase consistency loss. We recorded multichannel singing voices on robots and produced a 10-channel dataset to test multichannel singing voice separation algorithms. The output of the proposed model was a set of singing voice masks that could be used to transform the magnitude and phase spectrogram of the mixture into the singing voice. The experimental results show that the proposed model achieved higher performance on multichannel singing voice separation with distortion.

## Supporting information

S1 File Pretrained models and code. (ZIP)
